# The Molecular Effects of Sulforaphane and Capsaicin on Metabolism upon Androgen and Tip60 Activation of Androgen Receptor

**DOI:** 10.3390/ijms20215384

**Published:** 2019-10-29

**Authors:** Catalina Carrasco-Pozo, Kah Ni Tan, Tayner Rodriguez, Vicky M. Avery

**Affiliations:** 1 Discovery Biology, Griffith Institute for Drug Discovery, Griffith University, Nathan, Queensland 4111, Australia; kahni.tan@griffith.edu.au (K.N.T.); t.rodriguez@griffith.edu.au (T.R.); v.avery@griffith.edu.au (V.M.A.); 2 CRC for Cancer Therapeutics, Griffith Institute for Drug Discovery, Griffith University, Nathan, Queensland 4111, Australia

**Keywords:** sulforaphane, capsaicin, androgen receptor, Tip60, glycolysis, hexokinase, pyruvate kinase

## Abstract

Androgen receptor (AR) stimulators, such as androgen and Tip60, play a pivotal role in prostatic carcinogenesis as androgen receptor signaling is critical for the growth and transformation of the prostate gland. Moreover, androgen and Tip60 promotes HIF-1α activation, involved in metabolic reprogramming by increasing glycolysis, a hallmark in cancer initiation and development. In this study we evaluated the effect of androgen and Tip60 stimulus in AR pathway activation and HIF-1α stabilization, in terms of proliferation and cell metabolism in androgen-sensitive LNCaP cells. The protective role of the bioactive compounds sulforaphane and capsaicin against the effect of these stimuli leading to pro-carcinogenic features was also addressed. Sulforaphane and capsaicin decreased nuclear AR, prostate specific antigen and Bcl-XL levels, and cell proliferation induced by androgen and Tip60 in LNCaP cells. These bioactive compounds prevented the increase in glycolysis, hexokinase and pyruvate kinase activity, and reduced HIF-1α stabilization induced by androgen and Tip60 in LNCaP cells. The protective role of sulforaphane and capsaicin on prostate cancer may rely on mechanisms involving the inhibition of Tip60, AR and HIF-1α effects.

## 1. Introduction

Prostate cancer is the most commonly diagnosed cancer in men and is the fifth most common cause of cancer deaths globally [1]. Prostatic carcinogenesis is initially androgen-dependent and mediated primarily through androgen receptor (AR) signaling [2]. The AR is an androgen-responsive transcription factor, a member of the nuclear hormone receptor family that plays a key role in the growth, development, homeostasis and transformation of the prostate gland [3]. Androgen deprivation therapy (ADT) is the first-line treatment of symptomatic metastatic prostate cancer and as a neoadjuvant therapy for radiation therapy [4]. In addition to several adverse events, disease progression in men treated with ADT or surgical castration occurs as a result of the development of androgen-independent prostate cancer cells [4]. Tip60 is a histone acetyltransferase involved in multiple cellular processes, including chromatin remodeling, gene transcription, DNA damage responses, and tumorigenesis [5]. Tip60 is an androgen receptor co-activator acting via direct acetylation of lysine residues within the KLKK motif of the receptor hinge region, which is overexpressed in aggressive cases of prostate cancer [6,7]. Thus, inhibition of Tip60 has potential as a therapeutic strategy in prostate cancer, especially at the castrate-resistant stage.

In contrast to normal differentiated cells, which rely on mitochondrial oxidative phosphorylation to supply the energy demand for cellular processes, most cancer cells rely on aerobic glycolysis instead, for energy and biomass production; a phenomenon known as the Warburg effect [8]. Cancer cells reprogram their metabolism towards the glycolysis to promote growth, survival, proliferation, and long-term maintenance [9]. Androgen has been shown to modulate glycolytic metabolism in prostate cancer by stimulating glucose consumption and glycolytic rate, which may represent a crucial event for prostate tumor development [10,11]. HIF-1α, which is increased in many forms of cancer and can be activated by Tip60 and androgen [12,13], modulates energy metabolism in cancer cells by inducing over-expression of glycolytic enzymes including hexokinase (HK) and pyruvate kinase (PK) [14,15,16]. Similarly, prostate-specific antigen (PSA), a serum marker for prostate cancer [17], and Bcl-XL, an anti-apoptotic protein promoting cell survival [18], are also up-regulated due to prostate cancer. The preventive and therapeutic effects of dietary sulforaphane, an isothiocyanate found in broccoli, against human prostate cancer have been demonstrated in vivo, but the underlying mechanism(s) is poorly understood [19]. The chemoprevention of sulforaphane in prostate cancer has been mostly attributed to its ability to modulate gene expression through Nrf2-antioxidant signaling and epigenetics changes [20]. In addition, sulforaphane has been shown to inhibit HIF-1α expression induced by hypoxia [21] and to decrease glycolysis [22] in in vitro models of castrate-resistant prostate cancer. Interestingly, the metabolic role of sulforaphane in androgen-dependent prostate cancer has not been addressed yet. Capsaicin, the principal pungent component in the fruits of plants from the genus *Capsicum*, has been shown to suppress the growth of human prostate carcinoma cells in vitro and in vivo [23]. The antiproliferative activity of capsaicin in cancer correlates with oxidative stress induction, apoptosis and autophagy [24,25] as well as with its ability to inhibit the mitochondrial electron transport chain [26]. Noteworthy, the antiproliferative effect of capsaicin in prostate cancer mediated by AR signaling or metabolism reprogramming has not been addressed yet. The aim of this study was to evaluate the effects of androgen stimulus and Tip60 overexpression on AR-dependent glycolytic alterations in prostate cancer. The protective roles of sulforaphane and capsaicin in AR- and Tip60-mediated metabolic alterations, as well as their antiproliferative activity, were also addressed in this study.

## 2. Results

### 2.1. Increased Tip60 Levels via Both Androgen Stimulus and Genetic Overexpression

**Figure 1:**
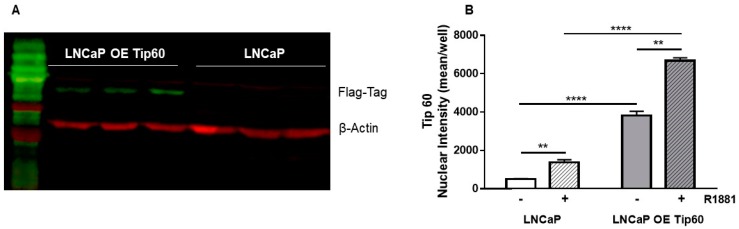
Tip60 levels are increased in LNCaP cells overexpressing Tip60 and exacerbated by androgen stimulus. (**A**) Tip60 levels were assessed by Flag-Tag detection through WB in LNCaP cells and in LNCaP cells overexpressing Tip60. This image is representative of three independent culture preparations, each treatment performed in triplicate. (**B**) Nuclear Tip60 levels in LNCaP cells and in LNCaP cells overexpressing Tip60, in the absence and presence of androgen (10 nM R1881, 72 h); detected by immunofluorescence using confocal imaging system and images were analyzed using Harmony software. Nucleus was identified through Hoechst staining. Values are expressed as mean ± SEM, from three independent culture preparations, each treatment performed in quadruplicate. Two-way ANOVA, Bonferroni post-test and p values comparisons are specified in the figure (** *p* < 0.01 and **** *p* < 0.0001). OE, overexpressing. R1881, synthetic androgen.

The levels of Tip60 in LNCaP cells and in Tip60 transduced LNCaP cells were evaluated through the measurement of Flag-Tag and Tip60 by immunofluorescence. Flag-Tag was only detected in Tip60 transduced LNCaP cells (Figure 1A) and its nuclear localization was increased by androgen stimulus (Supplementary Figure S3A,B). Androgen stimulus doubled the nuclear Tip60 levels in both LNCaP cells and in LNCaP cells overexpressing Tip60 (Figure 1B). 

### 2.2. Androgen Stimulus and Tip60 Overexpression Increased the Levels of Nuclear AR and Cytosolic PSA in LNCaP Cells

**Figure 2:**
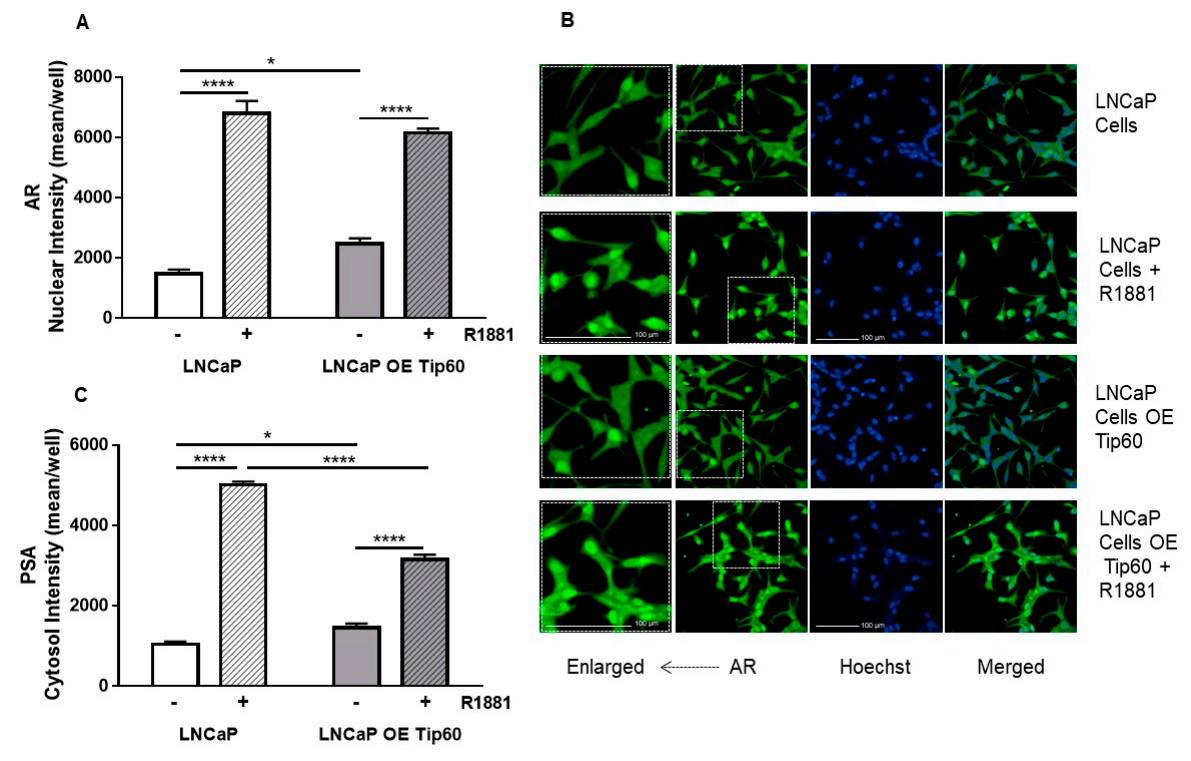
Nuclear AR and cytosolic PSA levels are increased by androgen stimulus and Tip60 overexpression in LNCaP cells. (**A**) Nuclear AR levels and (**B**) images, and (**C**) cytosolic PSA levels in LNCaP cells and in LNCaP cells overexpressing Tip60, in the absence or presence of androgen (10 nM R1881, 72 h). AR levels were detected by immunofluorescence using confocal imaging system. Images were acquired with 20x objective. Staining intensity levels in the nuclear region were obtained using Harmony software. Nucleus was identified through Hoechst staining. Scale is shown as 100 μm. White dotted frames indicate the section of the image that was enlarged. Values are expressed as mean ± SEM, from three independent culture preparations, each treatment performed in quadruplicate. Two-way ANOVA, Bonferroni post-test and p values comparisons are specified in the figures (* *p* < 0.05 and **** *p* < 0.0001). AR, androgen receptor; OE, overexpressing; R1881, synthetic androgen.

To evaluate the role of androgen and Tip60 overexpression in AR activation, the nuclear localization of AR and intracellular PSA levels were measured by immunofluorescence. As anticipated, androgen stimulus increased AR localization to the nucleus by 4.6-fold and cytosolic PSA levels by 4.8 in LNCaP cells (Figure 2A–C and Supplementary Figure S4). Tip60 overexpression by itself (in the absence of androgen) increased the AR and PSA levels by 68% and 40% in LNCaP cells (Figure 2A–C and Supplementary Figure S4). In LNCaP cells overexpressing Tip60, androgen stimulus increased the levels of AR by 2.5-fold and PSA by 2.2-fold.

### 2.3. Androgen Stimulus and Tip60 Overexpression Promoted Cell Proliferation and Increased Cytosolic Bcl-XL Levels

**Figure 3:**
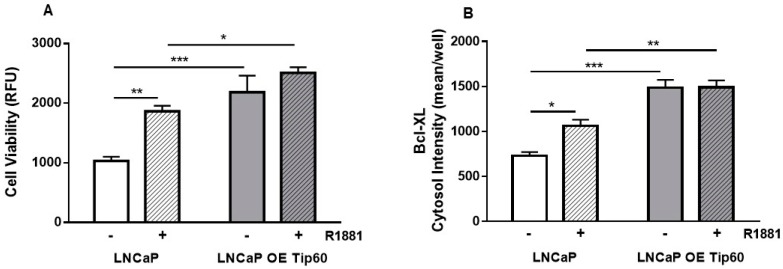
Cell proliferation and Bcl-XL levels are increased by androgen stimulus and Tip60 overexpression. (**A**) Cell proliferation and (**B**) cytosolic Bcl-XL levels of LNCaP cells and LNCaP cells overexpressing Tip60, in the absence or presence of 10 nM R1881 for72 h. The cell viability was assessed through their ability to reduce resazurin after 3 days of cell seeding. Bcl-XL levels were detected by immunofluorescence using confocal imaging system. Images were acquired with 20x objective. Staining intensity levels in the cytosolic region were obtained using Harmony software. Cytosol was identified through CellMask staining. Values are expressed as mean ± SEM, from three independent culture preparations, each treatment performed in quadruplicate. Two-way ANOVA, Bonferroni post-test and p values comparisons are specified in the figures (* *p* < 0.05, ** *p* < 0.01 and *** *p* < 0.001). OE, overexpressing; R1881, synthetic androgen.

The role of androgen and Tip60 overexpression in LNCaP cell proliferation was studied through the ability of live cells to reduce resazurin to resorufin, a red fluorescence dye. In addition, the effects of these stimuli in anti-apoptosis were assessed through the evaluation of Bcl-XL levels using immunofluorescence. After 3 days of culturing, Tip60 overexpression and androgen stimulus increased the proliferation of the LNCaP cells by 100% and 80%, respectively (Figure 3A and Supplementary Figure S1A–C). Tip60 overexpression increased the levels of the anti-apoptotic marker, Bcl-XL, by 41% in LNCaP cells (Figure 3B and Supplementary Figure S5). In LNCaP cells overexpressing Tip60, androgen stimulation had no effect on cell proliferation and Bcl-XL levels (Figure 3A,B, Supplementary Figures S1B,C and S5).

### 2.4. Androgen Stimulus and Tip60 Overexpression Increased Glycolysis and the Activity of Glycolytic Enzymes

**Figure 4:**
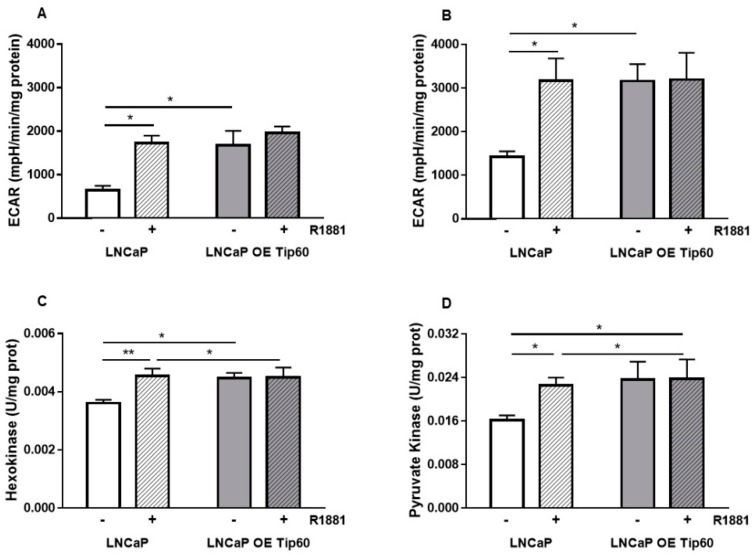
Glycolysis and the activity of glycolytic enzymes are increased by androgen stimulus and Tip60 overexpression. (**A**) Glycolysis, (**B**) glycolytic capacity, activities of (**C**) HK and (**D**) PK of LNCaP cells and LNCaP cells overexpressing Tip60, in the absence or presence of androgen (10 nM R1881, 72 h). Glycolysis and glycolytic capacity were assessed by measuring the extracellular acidification rate (ECAR) using Extracellular Flux Analyzer (Seahorse). ECARs were reported as mpH/min/mg protein. Activities of HK and PK were expressed as unit/mg protein. For HK, a unit was calculated as nmol of NADP reduced/min. For PK, a unit was calculated as nmol of NADH oxidized/min/mg protein. Values are expressed as mean ± SEM, from three independent culture preparations, each treatment performed in triplicate. Two-way ANOVA, Bonferroni post-test and p values comparisons are specified in the figures (* *p* < 0.05 and ** *p* < 0.01). OE, overexpressing; R1881, synthetic androgen.

The effect of androgen and Tip60 overexpression in glycolysis was studied through the quantification of the extracellular acidification rate, using an Extracellular Flux Analyzer (Seahorse). Androgen stimulus and Tip60 overexpression both increased the glycolysis and glycolytic capacity of the cells by 65% and 73% in LNCaP cells, respectively (Figure 4A,B). 

The role of androgen and Tip60 overexpression in the activity of glycolytic enzymes was also studied. HK activity was evaluated by determining the reduction rate of NADP at 340 nm, in the presence of its substrate, glucose. The activity of PK was assessed by the oxidation rate of NADH at 340 nm, in the presence of phosphoenolpyruvate. Androgen stimulus and Tip60 overexpression also increased the activity of HK and PK by 26% and 40% in LNCaP cells (Figure 4D,E). Androgen stimulus did not further increase the glycolysis and glycolytic enzymes in LNCaP cells overexpressing Tip60 (Figure 4A–D). 

### 2.5. Androgen Stimulus and Tip60 Overexpression Increased HIF-1α Levels

**Figure 5:**
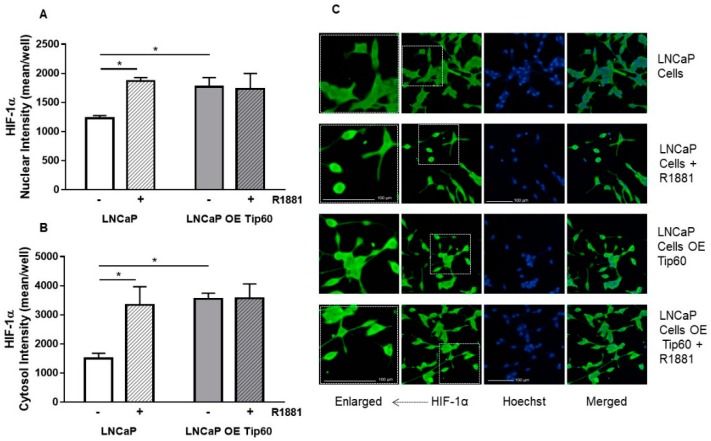
HIF-1α levels are increased by androgen stimulus and Tip60 overexpression in LNCaP cells. (**A**) Nuclear and (**B**) cytosolic HIF-1α levels and (**C**) images in LNCaP cells and in LNCaP cells overexpressing Tip60, in the absence or presence of androgen (10 nM R1881, 72 h). HIF-1α levels were detected by immunofluorescence using confocal imaging system. Images were acquired with 20x objective. Staining intensity levels in the nucleus and cytosolic region were obtained using Harmony software. Nucleus and cytosol were identified through Hoechst and CellMask staining, respectively. Scale is shown as 100 μm. White dotted frames indicate the section of the image that was enlarged. Values are expressed as mean ± SEM, from three independent culture preparations, each treatment performed in quadruplicate. Two-way ANOVA, Bonferroni post-test and p values comparisons are specified in the figures (* *p* < 0.05). HIF-1α, hypoxia-inducible factor-1α; OE, overexpressing; R1881, synthetic androgen.

The effect of androgen and Tip60 overexpression on LNCaP intracellular HIF-1α levels was studied using immunofluorescence. Androgen stimulus increased HIF-1α levels by 52% in the nucleus and 2.2-fold in the cytosol of LNCaP cells, whereas LNCaP cells overexpressing Tip60 had increased HIF-1α levels by 44% in the nucleus and 2.4-fold in the cytosol compared to LNCaP cells (Figure 5A–C). Androgen stimulus did not further increase the HIF-1α levels in LNCaP cells overexpressing Tip60 (Figure 5A–C). 

### 2.6. Sulforaphane and Capsaicin Inhibited the Increase of Nuclear AR and Cytosolic PSA Induced by Androgen Stimulus and Tip60 Overexpression

**Figure 6:**
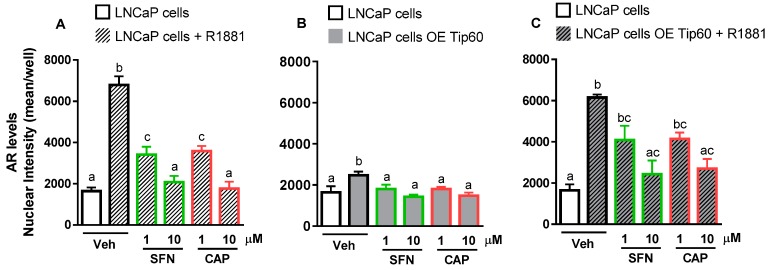
The increase of nuclear AR levels induced by androgen and Tip60 overexpression in LNCaP cells are inhibited by sulforaphane and capsaicin. Nuclear AR levels in (**A**) LNCaP cells + androgen stimulus, (**B**) LNCaP cells overexpressing Tip60 and (**C**) LNCaP cells overexpressing Tip60 + androgen stimulus, in comparison to the basal levels of LNCaP cells treated only with vehicle (Veh, 0.4% DMSO). Cells were treated with the compounds added as in Scheme 1. AR levels were detected by immunofluorescence using confocal imaging system. Images were acquired with 20x objective. Staining intensity levels in the nuclear region were obtained using Harmony software. Nucleus was identified through Hoechst staining. Values are expressed as mean ± SEM, from three independent culture preparations, each treatment performed in quadruplicate. For all bars with the same letter, the difference between the means is not statistically significant. Values with different letters indicate significant differences (*p* < 0.05) between bars (one-way ANOVA, Bonferroni post-test). CAP, capsaicin; OE, overexpressing; SFN, sulforaphane.

Sulforaphane and capsaicin prevented the increase induced by androgen stimulus (Figure 6A), Tip60 overexpression (Figure 6B) and both androgen and Tip60 stimuli (Figure 6C) in LNCaP cells. At 1 µM sulforaphane and capsaicin fully inhibited the increase in AR levels induced by Tip60 (Figure 6A), while at 10 µM they completely inhibited the increase induced by androgen, either in LNCaP cells (Figure 6A) or LNCaP cells overexpressing Tip60 (Figure 6C). 

At a concentration of 10 µM, sulforaphane and capsaicin completely prevented the increase of PSA levels either induced by androgen (Supplementary Figure S6A), Tip60 overexpression (Supplementary Figure S6B) or both androgen and Tip60 stimuli (Supplementary Figure S6C) in LNCaP cells. Sulforaphane and capsaicin had no effect on the basal levels of AR and PSA in LNCaP cells in the absence of any stimulus (Supplementary Figure S7A,B). 

### 2.7. Sulforaphane and Capsaicin Inhibited the Increase in Bcl-XL Levels Induced by Androgen Stimulus and Tip60 Overexpression

**Figure 7:**
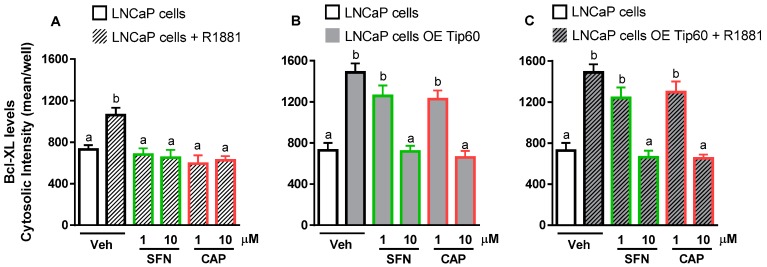
The increase of Bcl-XL levels induced by androgen and Tip60 overexpression in LNCaP cells are inhibited by sulforaphane and capsaicin. Cytosolic levels in (**A**) LNCaP cells + androgen (10 nM R1881), (**B**) LNCaP cells overexpressing Tip60 and (**C**) LNCaP cells overexpressing Tip60 + androgen stimulus, in comparison to the basal levels of LNCaP cells treated only with vehicle (Veh, 0.4% DMSO). Cells were treated with the compounds as in Scheme 1. Bcl-XL levels were detected by immunofluorescence using confocal imaging system, with 20x objective. Cytosol was identified through CellMask staining and staining intensity levels were obtained using Harmony software. Values are expressed as mean ± SEM, from three independent culture preparations, each treatment performed in quadruplicate. For all bars with the same letter, the difference between the means is not statistically significant. Values with different letters indicate significant differences (*p* < 0.05) between bars (one-way ANOVA, Bonferroni post-test). CAP, capsaicin; OE, overexpressing; SFN, sulforaphane.

Sulforaphane and capsaicin at 1 µM totally prevented the increase in the cytosolic levels of the anti-apoptotic biomarker, Bcl-XL induced by androgen (Figure 7A). At 10 µM sulforaphane and capsaicin, completely inhibited the increase in the antiapoptotic biomarker induced by Tip60, either in the absence (Figure 7B) or presence of androgen (Figure 7B). Sulforaphane and capsaicin had no effect on the basal levels of cytosolic Bcl-XL in LNCaP cells (Supplementary Figure S7C).

### 2.8. Sulforaphane and Capsaicin Reduced Glycolysis and Glycolytic Enzyme Activities in LNCaP Cells Overexpressing Tip60 Stimulated with Androgen

Increased glycolysis and related enzyme activities were attained to the same extent with any of the tested stimuli, with no further increase observed when both stimuli were present (Figure 5). Therefore, the effects of the compounds were assessed in LNCaP cells overexpressing Tip60 in the presence of androgen. 

**Figure 8:**
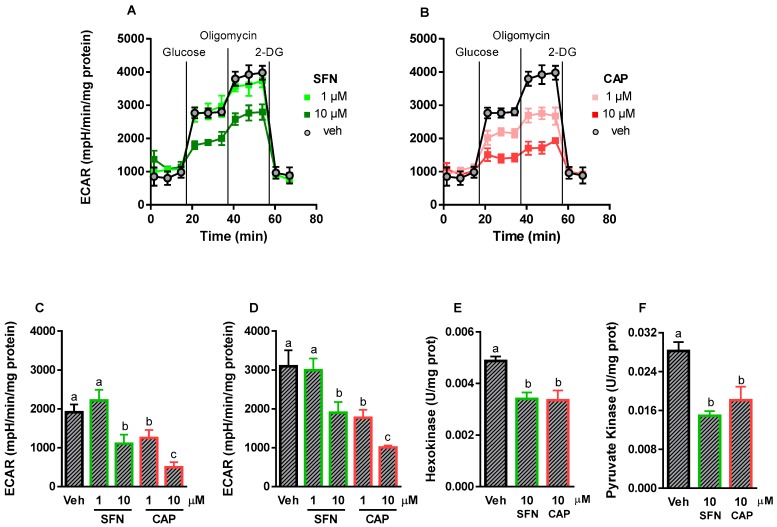
The increase of glycolysis, glycolytic capacity and glycolytic enzyme activities in androgen-stimulated LNCaP cells overexpressing Tip60 is reduced by sulforaphane and capsaicin. Extracellular acidification rate (ECAR) in LNCaP cells overexpressing Tip60 incubated with androgen for 72 h (10 nM R1881) and treated with (**A**) sulforaphane or (**B**) capsaicin (compounds added every 24 h for 3 days as in Scheme 1). ECAR was measured using Extracellular Flux Analyzer (Seahorse) and expressed as mpH/min/mg protein. (**C**) Glycolysis and (**D**) glycolytic capacity were quantified from the ECAR curves. Values are expressed as mean ± SEM, from three independent culture preparations, each treatment performed in duplicate at least. Activities of (**E**) HK and (**F**) PK in LNCaP cells overexpressing Tip60 incubated with androgen for 72 h (10 nM R1881), treated with sulforaphane or capsaicin. Activities of HK and PK were expressed as unit/mg protein. For HK, a unit was calculated as nmol of NADP reduced/min. For PK, a unit was calculated as nmol of NADH oxidized/min/mg protein. Values are expressed as mean ± SEM, from three independent culture preparations, each treatment performed in quadruplicate. For all bars with the same letter, the difference between the means is not statistically significant. Values with different letters indicate significant differences (*p* < 0.05) between bars (one-way ANOVA, Bonferroni post-test). CAP, capsaicin; SFN, sulforaphane.

Sulforaphane at 10 µM reduced the glycolysis and glycolytic capacity by 42% and 39%, respectively (Figure 8A,C,D). Capsaicin reduced the glycolysis and glycolytic capacity in a concentration-dependent manner and at 10 µM reduced the glycolysis by 74% and glycolytic capacity by 67% (Figure 8B–D). 

Sulforaphane and capsaicin at 10 µM reduced the HK activity by 30% (Figure 8E) in LNCaP cells overexpressing Tip60 stimulated by androgen. In addition, sulforaphane and capsaicin at the same concentration reduced the PK activity by 47% and 34%, respectively in these cells (Figure 8F). 

### 2.9. Sulforaphane and Capsaicin Reduced the Increased HIF-1α Levels Induced by Androgen Stimulus and Tip60 Overexpression

**Figure 9:**
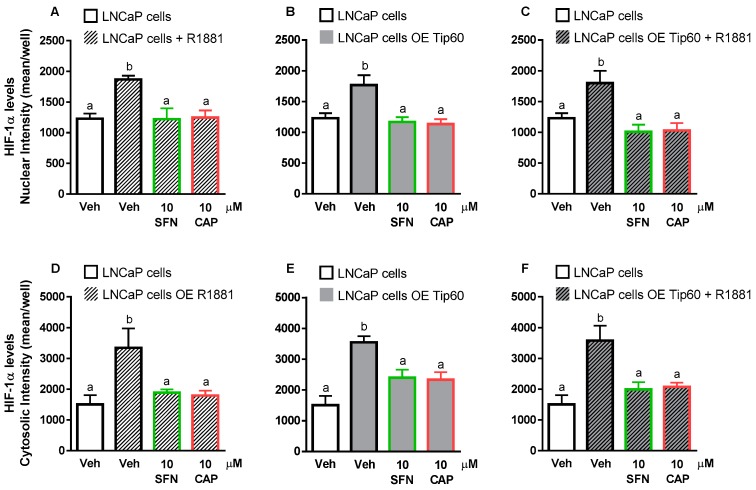
The increase of HIF-1α levels in LNCaP cells overexpressing Tip60 androgen stimulated is inhibited by sulforaphane and capsaicin. Nuclear (**A**–**C**) and cytosolic (**D**–**F**) HIF-1α levels in (**A**,**D**) LNCaP cells + androgen stimulus, (**B**,**E**) LNCaP cells overexpressing Tip60 and (**C**,**F**) LNCaP cells overexpressing Tip60+ androgen stimulus, in comparison to the basal levels of LNCaP cells treated only with vehicle (Veh, 0.4% DMSO). Compounds were added every 24 h for 3 days (Scheme 1). HIF-1α levels were detected by immunofluorescence using confocal imaging system at 20x objective. Staining intensity levels in the nuclear and cytosolic region were obtained using Harmony software. Nucleus and cytosol were identified through Hoechst and CellMask staining, respectively. Values are expressed as mean ± SEM, from three independent culture preparations, each treatment performed in quadruplicate. For all bars with the same letter, the difference between the means is not statistically significant. Values with different letters indicate significant differences (*p* < 0.05) between bars (one-way ANOVA, Bonferroni post-test). CAP, capsaicin; OE, overexpressing; SFN, sulforaphane.

Sulforaphane and capsaicin at 10 µM completely prevented the increase in nuclear and cytosolic HIF-1α levels induced by androgen (Figure 9A,D), Tip60 (Figure 9B,E) and both stimuli (Figure 9C,F) in LNCaP cells. These compounds did not alter either the nuclear or cytosolic HIF-1α levels in non-stimulated LNCaP cells (Supplementary Figures S7D and S6E).

## 3. Discussion

Androgen receptor stimulators, such as androgen and Tip60, play a pivotal role in prostatic carcinogenesis as androgen receptor signaling is critical for the growth and transformation of the prostate gland [2]. Moreover, androgen and Tip60 promote HIF-1α activation [12,13], shown to promote metabolic reprogramming by increasing glycolysis [16], a hallmark of cancer initiation and development. In this study, we found that sulforaphane and capsaicin prevented the activation of AR signaling (decreased nuclear AR levels and PSA levels) and glycolysis (decreased EACR; and HK and PK activities) induced by androgen and Tip60. 

### 3.1. Androgen Stimulus and Tip60 Activate AR Signalling and Increase Proliferation

Our results demonstrated that androgen and Tip60 activate the AR pathway, as evidenced by the increase in nuclear AR levels (Figure 2A,B) and cytosolic PSA levels (Figure 2C and Supplementary Figure S4). Consistent with previous findings that AR signalling is involved in cell proliferation, we found that androgen-sensitive LNCaP cells did not proliferate in the absence of androgen (Figure 3A and Supplementary Figure S1A). In line with the crucial role of Tip60 in LNCaP cell proliferation, we found that Tip60 overexpression increased cell growth in LNCaP cells even in the absence of androgen (Figure 3A and Supplementary Figure S1C).

It has been reported that an acetylated phenotype of AR exhibits an augmented proliferation in androgen-independent LNCaP cell [27]. Moreover, androgen-induced LNCaP cell proliferation is reduced when Tip60 is downregulated and silencing of Tip60 decreases proliferation of CxR cells, an androgen-insensitive LNCaP derivative cell line [27]. These findings indicate that the effect of the inhibition of Tip60 on cell proliferation is AR-mediated, either in androgen-sensitive or-insensitive cells; whether in the presence or absence of androgen. 

The ability of androgen to induce the anti-apoptotic biomarker Bcl-XL, via AR receptor activation, is the mechanism underlying the increased cell proliferation (Figure 3B and Supplementary Figure S5). In fact, blocking Bcl-XL expression in prostate cancer cells decreased cell proliferation [28,29] and silencing the AR gene in prostate cancer cells significantly reduced Bcl-XL expression and increased apoptosis [30]. Recently, it has been shown that K104 acetylation of Tip60 plays an essential role in inducing apoptosis upon glucose starvation via p53 activation in hepatocellular cancer cells [31]. In contrast, we found that Tip60 increased Bcl-XL expression without-glucose deprivation (Figure 3B and Supplementary Figure S5), indicating that Tip60 has a double-edged role in apoptosis, that of triggering different pathways depending on limited nutrient supply and metabolic stress. 

### 3.2. Androgen and Tip60 Stimulus Stabilize HIF-1α and Increase Glycolysis

Glycolysis is considered the major metabolic process for energy production and anabolic growth in cancer cells. Our results showed that androgen and Tip60 both independently increase glycolysis (Figure 4A,B) and glycolytic enzyme activities, such as HK and PK (Figure 4C,D), which correlates with the metabolic adaptations cancer cells undergo to sustain enhanced cell proliferation (Figure 3A and Supplementary Figure S1C). HK-II, the predominant isoform of HK in cancer, promotes glycolysis by suppressing the negative feedback induced by glucose-6-phosphate [32]. In addition, HK-II inhibits apoptosis as its interaction with the voltage-dependent anion channel (VDAC) at the mitochondrial membrane inhibits the release of the pro-apoptotic biomarkers [33]. Bcl-XL, by increasing the affinity of VDAC to HK, contributes to the anti-apoptotic role of the enzyme [34]. Increased HK activity has been proposed as a mechanism of action for androgen to increase glucose utilization through glycolysis [10]. 

Importantly, we have demonstrated for the first time that Tip60 overexpression is sufficient to upregulate the activities of HK and PK in prostate cancer cells in the absence of androgen, thus contributing to the increase in glycolysis (Figure 4D). Tip60 has been shown to acetylate PKM2, a highly expressed isoform in cancer, in HeLa cells co-transfected with Tip60 and PKM2 [35] and that p300, another acetyltransferase, acetylates/promotes PKM2 activity [36]. Moreover, PK has been shown to promote glycolysis by inactivating pyruvate dehydrogenase, the enzyme that catalyzes the conversion of pyruvate to acetyl-CoA, a rate-limiting step of entry into the mitochondria and the tricarboxylic cycle [37].

HIF-1α, an essential transcription factor that mediates energy metabolism under hypoxic conditions, has been shown to promote glycolysis by upregulating the transcription of glycolytic enzymes, such as HK and PK [16,38]. The role of Tip60 in HIF-1α activation has been implicated in hypoxia based on the expression of HIF-1α-dependent genes in colorectal cancer cells [12]. Here we showed that Tip60 overexpression stabilizes HIF-1α in LNCaP cells under normoxia, as evidenced by increased protein levels not only in the nucleus, but also in the cytosol (Figure 5A–C) independent of androgen stimulus. Under normoxic conditions, HIF-1α protein is rapidly degraded by the ubiquitin protein ligase Von Hippel-Lindeau (VHL) in the proteosome, thus very little HIF-1α is detectable under normoxic conditions [39]. In addition, we observed that HIF-1α can also be stabilized by androgen, in fact it has been shown that androgen does not regulate HIF-1α mRNA transcription, but block HIF-1α protein degradation in LNCaP cells, mimicking what occurs during hypoxia [13]. These findings suggest that androgen and Tip60 via stabilizing HIF-1α under normoxia, promote the expression of glycolytic enzymes, which contribute to an increase in glycolysis.

### 3.3. Sulforaphane and Capsaicin Inhibited AR Pathway and Proliferation Induced by Androgen and Tip60 Overexpression

We found that sulforaphane and capsaicin reduced nuclear AR levels (Figure 6A–D) and cytosolic PSA levels (Supplementary Figure S6A–C) induced by androgen and Tip60. Both compounds normalize AR and PSA levels to the same extent as non-androgen stimulated LNCaP cells. These results indicate that Tip60 is involved in the AR pathway activation, independent of androgen stimulus. In fact, it has been shown that downregulation of Tip60 or Tip60-defective mutant decreases AR activation and reduces PSA mRNA levels induced by androgen in LNCaP cells or in androgen-insensitive prostate cancer cells [6,27].

We found that 10 μM sulforaphane co-incubated with androgen for 72 h inhibited AR activation, which has also been found with shorter incubation time (24 h) with similar dose of sulforaphane in LNCaP cells in the presence [40] or absence of androgen [41]. Sulforaphane (20 μM) has been shown to decrease AR mRNA level after 24 h incubation in androgen-starved cells [40] and to induce AR degradation, by inactivating histone deacetylase 6, after 12 h incubation in androgen-stimulated LNCaP cells [42]. As we did not observe a decrease in nuclear AR protein levels after 72 h treatment in androgen-starved LNCaP cells (Figure 6A), the effect of sulforaphane on AR expression and degradation seems to be transient and cells may undergo adaptation under the repetitive sulforaphane dosing that we administered. 

Short–term exposure of capsaicin at high doses (100–500 µM, 24 h) to LNCaP cells has been shown to inhibit the transcription of PSA not only via decreasing AR nuclear levels, but also by a direct inhibitory effect on PSA transcription [43,44]. Consistently, we also found in our studies that capsaicin inhibited the AR pathway in LNCaP cells at a lower concentration (10 μM) as a consequence of longer and repetitive exposure. 

Although it is known that sulforaphane and capsaicin can reduce LNCaP cell proliferation [40,41,43,44], this is the first time that the sensitivity of this cell line to the cytotoxic effects of these agents has been shown to be influenced by the presence of androgen and Tip60 (Supplementary Figure S2A–D and Supplementary Table S1). As higher concentrations of compounds are needed to decrease the proliferation of cells with androgen stimulus and/or Tip60 overexpression, it is suggested that sulforaphane and capsaicin do not inhibit proliferation by targeting the cell division. These compounds could be strategic adjuvants in chemotherapy by acting through complimentary mechanism to reduce cell expansion in prostate cancer, such as inhibiting AR pathway.

It has been shown that Bcl-XL [45], but not Bcl-2 overexpression, another anti-apoptotic biomarker [46], protects prostate cancer cells against sulforaphane-induced cell death, indicating that Bcl-XL plays a crucial role in sulforaphane’s mechanism for cell damage. We suggest that androgen stimulus and Tip60 overexpression confer more resistant to the inhibition of LNCaP cells proliferation induced by sulforaphane and capsaicin by a mechanism involving increased Bcl-XL level (Figure 3B and Supplementary Figure S5). Consistent with our findings, the effects of sulforaphane in reducing Bcl-XL and decreasing cell viability have been shown in hepatocarcinoma HepG2 cells [47] and PC3 cells [48]. Interestingly, only at 50 µM, were sulforaphane and capsaicin able to increase DNA fragmentation, and only by 20%, as determined by Hoechst aggregation (data not shown). This suggests the compounds impacted on apoptosis and cell proliferation through their action against HK and Bcl-XL.

### 3.4. Role of Sulforaphane and Capsaicin Inhibited HIF-1α Stabilization Induced by Androgen and Tip60 Overexpression

We observed that sulforaphane and capsaicin reduced HIF-1α levels increased by androgen stimulation and Tip60 overexpression, in the nucleus and cytosol (Figure 9A–H), which suggests a mechanism involving HIF-1α stability. In fact, it has been shown that sulforaphane decreases hypoxia-increased HIF-1α half-life in HCT116 colon cancer cells [49] and that capsaicin induces p53-mediated HIF-1α degradation in non-small cell lung carcinoma cells in hypoxic environment [50]. 

We suggest that sulforaphane and capsaicin decreased glycolysis (Figure 8A–D) induced by androgen and Tip60 overexpression via altering HIF-1α stability and thus, decreasing its transcriptional activity. Reduction in HK and PK protein levels may be manifested by decreased HK and PK enzyme activities which were observed in LNCaP cells overexpressing Tip60 treated with androgen (Figure 8E,F). In fact, sulforaphane has been shown to decrease HK-II and PKM2 levels in breast cancer cell lines [51] and 6-phosphofructo-2-kinase/fructose-2,6-biphosphatase4 (PFKFB4) via reducing HIF-1α expression in hepatocellular carcinoma cells [52]. PFKFB4 is one of the four isoforms of the bifunctional kinase and phosphatase enzyme PFK2 (6-phosphofructo-2-kinase/fructose-2,6-bisphosphatase) and plays a key role in glycolysis and gluconeogenesis in cancer [53]. Capsaicin has been shown to inhibit glycolysis in oesophageal squamous cell carcinoma cells, which was associated with a decreased HK-II expression [54].

### 3.5. In Vivo Significance of the Health Effects of Sulforaphane and Capsaicin in Terms of Their Effective Concentrations 

Although the concentration of sulforaphane used in our study cannot be reached in vivo through consumption of broccoli in a normal diet, it could be attained through the intake of a diet rich in myrosinase-treated broccoli or sulforaphane-based nutraceuticals. Myrosinase is a temperature sensitive enzyme that converts glucoraphanin from broccoli into sulforaphane [55]. In fact it has been shown that increased sulforaphane bioavailability can be attained after the intake of sulforaphane-enriched broccoli sprout preparation (generated by quick steaming followed by myrosinase treatment) in mice [56]. 

It is unlikely that the effective concentration of capsaicin studied in vitro here can be reached in vivo by the diet, however it could be reached by the oral intake of encapsulated capsaicin. Liposomal and methoxypoly (ethylene glycol)-poly(ε-caprolactone) microencapsulation increase capsaicin bioavailability by 3.34-fold and 6-fold respectively in rats [57,58]. In addition, considering that the minimum lethal oral dose of capsaicin is 100 mg/Kg body weight in mice, its consumption could be safely increased [59]. 

## 4. Materials and Methods 

### 4.1. Culturing Conditions and Assay Format

**Scheme 1:**
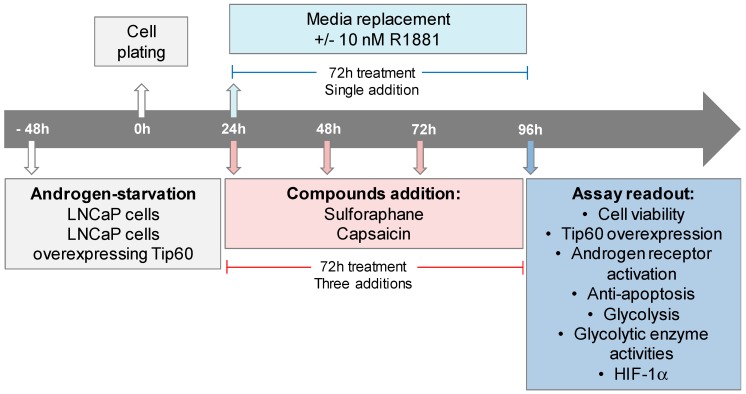
Assay format and compound addition format.

LNCaP cells clone FGC (ATCC^®®^ CRL-1740™ passage 7–20) were cultured in RPMI media (Cat# 11875093, Life Technologies, Carlsbad, CA, USA) supplemented with 10% FBS Australia Source (Cat# 35-076-CV, Corning, Woodland, CA, USA) LNCaP cells overexpressing Tip60 were cultured in RPMI media supplemented with 10% FBS and 1.2 mg/mL geneticin (Cat# 10131035, Gibco, Waltham, MA, USA). Cells were maintained at 37 °C in a humidified incubator with 5% CO_2_. Cells were starved of androgens in RPMI media without phenol red (Cat# 11835030, Life Technologies,) supplemented with 10% charcoal-stripped FBS Australia Source (Cat# 12676029, ThermoFisher, Waltham, MA, USA) for 72 h prior to any treatment (Scheme 1). After 24 h of seeding, cells were treated with 10 nM of the synthetic androgen, R1881, as at this concentration it stimulated LNCaP cell (Supplementary Figure S1A). Cells were treated with compounds as follows: sulforaphane (Cat# S4441, Sigma Aldrich, St. Louis, MO, USA), capsaicin (Cat# 211274, Calbiochem, Darmstadt, Germany) or compound vehicle (negative control, 0.4% DMSO). Compounds and vehicle were added three times, after 24 h, 48 h and 72 h of seeding the cells (Scheme 1). Measurements were made after 24 h of the last addition of compound or vehicle. The compounds were subsequently evaluated using concentrations equivalent to their IC_50_ (sulforaphane and capsaicin = 1 μM) and IC_10_ (sulforaphane and capsaicin = 10 μM) values previously determined in the 72 h proliferation assay with LNCaP cells without stimulus. (Supplementary Figure S2A and Supplementary Table S1). In the proliferation assay compounds were evaluated in a range of concentrations between 0.06 to 200 μM to obtain a concentration response curve.

### 4.2. LNCaP Cells Tip60 Overexpression

LNCaP clone FGC (ATCC^®®^ CRL-1740™) was infected with purified lentiviral particles for Tip60 (NM_006388.3) with 3 × Flag-Tag at the N terminal (Cat# LPP-CS-S0359-Lv151-01-200, GeneCopoeia, Inc, Rockville, MD, USA) LNCaP cells were plated into a 24-well plate (Cat# 662160, Greiner Bio-One, Monroe, NC, USA) at a density of 30,000 cells per well prior to viral infection, thus cells reached 70–80% confluency at the time of transduction. After 24 h, growth media was removed from the wells and replaced with 500 µL of complete media with polybrene (hexadimethrine bromide) from Sigma-Aldrich (Cat# H9268-5G) at a concentration of 8 μg/mL. A multiplicity of infection of 5 was used with a viral titer of 2.68 × 10^8^ TU/mL, and uninfected wells were used as a standard control. The following day culture medium was removed and replaced with 500 µl of complete medium per well and cells were incubated at 37 °C with 5% CO_2_ for a further 48 h. The antibiotic selection started at 0.5 mg/mL with geneticin and increasing to 1.2 mg/mL. Single drug-resistant colonies were selected and maintained at 1.2 mg/mL geneticin. Selected cells were expanded for 3 weeks in T25 (25 cm²), then for 4 weeks in T75 (75 cm²) and finally working stocks were grown on T175 flasks (175 cm²) for 3 weeks. 

### 4.3. Cell Proliferation Rate

Cells were seeded at 2500 cells/well/50 µL in 384-well plate (Cat# 781090, Greiner 384 Plates Black) and the media replaced with phenol red-free RPMI media supplemented with 10% charcoal stripped FBS +/- 10 nM R1881 (Cat# R0908, Sigma Aldrich,) every 2 days. At 3, 6, 7 and 10 days post seeding, cells were incubated with 60 µM resazurin (Cat# 14322, Cayman, Ann Arbor, MI, USA) for 6 h and the reduction of the dye was detected by fluorescence at λ_excitation_ 530 nm; λ_emission_ 590 nm, using a Microplate Reader (EnSpire, Perkin Elmer, Baesweiler, Germany). Background readings from wells that contain media and resazurin only were subtracted from the relative fluorescent unit (RFU) of each sample well. 

### 4.4. Cell viability Inhibition

After 24 h cell seeding, the media was replaced with phenol red-free RPMI media supplemented with 10% charcoal stripped FBS ± 10 nM R1881. Cells were treated with sulforaphane, capsaicin or 0.4% DMSO according to Scheme 1. Following 24 h incubation after final compound addition, RFU of resazurin reduction were measured and % of inhibition was calculated in relation to the positive control (30 µM puromycin, Cat# ant-pr-1, InvivoGen, San Diego, CA, USA) as: ((RFU_0.4% DMSO_ − RFU_sample_) x 100)/(RFU_0.4% DMSO_ − RFU_puromycin_). Fluorescence intensity values were normalized to the negative and positive control. Dose-response curves (log(inhibitor) vs response, four parameter variable slope) were obtained using Graphpad Prism 7.0 (GraphPad Inc., La Jolla, CA, USA). 

### 4.5. Western Blot

Western blots were used to confirm Tip60 overexpression by detecting Flag-Tag signal levels. One × 10^6^ cells from the expanded drug-resistant colonies were lysed in 1× lysis buffer which was prepare with 10× Cell Lysis Buffer (Cat# 9803S, Cell Signalling, Danvers, MA, USA), complete™ Mini EDTA-free protease inhibitor cocktail (Cat# 11836170001, Roche, Basel, Switzerland) and phosphatase inhibitor cocktail PhosSTOP EASYpack (Cat# 4906845001, Roche). Protein estimations were done using the DC™ Protein Assay Kit (Cat# 5000111, Bio-Rad, Hercules, CA, USA). Gels were run at 120 V for 65 min; PVDF membranes, filter papers and gels equilibrated with the respective buffers and a semi-dry transfer performed using the Trans-Blot Turbo Transfer System (Bio-Rad) at 25 V for 25 min. Blots were blocked with Tris-buffered saline (TBS) (Cat# 927-60001, LI-COR, Lincoln, NE, USA) for 1 h at room temperature. Blots were incubated overnight at 4 °C with DYKDDDDK 3× Flag-Tag (D6W5B) (1/1000, rabbit monoclonal antibody (mAb), Cat#:14793, Cell Signalling), β-Actin (1/1500, mouse mAb, Cat#: A2228, Sigma-Aldrich) prepared in 0.1% Tween20 in blocking buffer. Secondary Ab infrared IRDye^®®^ 800CW donkey (polyclonal) anti-rabbit IgG (H + L) (1/15000, Cat#: 926-32213, LI-COR) and IRDye^®®^ 680RD donkey (polyclonal) anti-mouse IgG (H + L) (1/15000, Cat#: 926-68072, LI-COR), were prepared in 0.1% Tween20 in TBS and incubated for 2 h at room temperature. Blots were developed using the Odyssey Fc Imaging System (LI-COR, Lincoln, NE, USA). 

### 4.6. Immunofluorescence

Cells were seeded at 2500 cells/well/50 µL in RPMI media supplemented with 10% FBS in a 384-well plate (CellCarrier Ultra, Cat # 6057300, PerkinElmer). After 24 h, media was replaced with phenol red-free RPMI media supplemented with 10% charcoal stripped FBS +/- 10 nM R1881 (R0908, Sigma Aldrich). After sulforaphane or capsaicin treatment (Scheme 1), cells were fixed in 4% paraformaldehyde (PFA, Cat# 28908, ThermoFisher) solution for 15 min and washed 3× with PBS (Cat# 14190250, ThermoFisher/Gibco). Cells were blocked for 2 h at room temperature with a buffer containing 2% BSA and 0.2% Triton-X 100, then incubated overnight at 4 °C with the primary Abs: AR (1/400, rabbit mAb, Cat # 5153, Cell Signaling), PSA (1/2000, rabbit polyclonal antibody (pAb), Cat # A056201-2, Dako Santa Clara, CA, USA), Bcl-xL (1/200 rabbit mAb, Cat# 2764, Cell Signaling), Tip60 (1/1000 rabbit pAb, Cat# PA5-34548, Thermo Fisher Scientific), HIF-1α (1/200, rabbit pAb, Cat# NB100-134, Novus Biologicals, Littleton, CO, USA) and DYDDDDK TAG (1/400, mouse mAb, Cat# 8146S, Cell Signaling) in PBS. After washing 3× with PBS, cells were incubated for 2 h at room temperature with Hoechst 33342 (10 μM, Cat# H1399, Life Technologies) to identify nucleus; HCS CellMask™ Deep Red Stain (0.2 μg/mL, Cat# H32721, Molecular Probe, Eugene, OR, USA to identify cytosol and the secondary Abs: Alexa Fluor 488 goat anti-rabbit IgG (H + L) (1/400, Cat# A11008, Thermo Fisher) or goat anti-mouse (1/400, Cat# A11001, Thermo Fisher) according to the primary Ab species and all prepared in PBS. Cells were washed 3× in PBS and imaged on the Opera Phenix™ High Content Screening System (Perkin Elmer, Baesweiler, Germany) using a 20× water objective and nine fields imaged per well. Hoechst was detected at 405 nm; Alexa Fluor 488 secondary antibodies at 488 nm and HCS CellMask™ Deep Red Stain detected at 655nm. The selected area was applied to calculate the fluorescence of Alexa Fluor 488 in the cytosol. Images were analyzed using Harmony software (Perkin Elmer, Baesweiler, Germany).

### 4.7. Enzyme Activity Assays

The activities of HK and PK were measured through continuous spectrophotometric assays as previously described [60]. After adding 20 μg of protein extract to the reaction mix (Supplementary Table S2), reactions were started by addition of the respective initiating substrates just prior to measurement using a Microplate Reader (EnSpire, Perkin Elmer). The linear portion of the reaction was used to determine the rate of change in absorbance and enzyme activity was then calculated and normalized to protein content. HK was calculated as nmol of NADP reduced/min/mg protein and PK was calculated as nmol of NADH oxidized/min/mg protein. Protein content was measured by the method of Bradford (Bio-Rad Protein Assay, Bio-Rad, Hercules, CA, USA). 

### 4.8. Extracellular Acidification Rates

Extracellular acidification rates (ECAR) were measured using the XFp Extracellular Flux analyzer (Seahorse Bioscience, North Billerica, MA, USA). Briefly, 15,000 cells/well were plated into XFp polystyrene cell culture plates (Seahorse Bioscience) coated with 0.1 mg/mL collagen (Cat# C-7521, Sigma). Cells were incubated for 72 h with the treatments as Scheme 1 in a humidified 37 °C incubator with 5% CO_2_. Media was replaced with XFp base media (at# SEA103193100C, Seahorse Bioscience) supplemented with 1 mM glutamine (pH 7.4) and cell plates were incubated in a 37 °C/non-CO_2_ incubator for 1 h prior to the start of an assay in the absence or presence of 100 mM oxamate, a lactate dehydrogenase (LDH) inhibitor [61]). 

Twenty μL of 20 mM glucose, 1 µM oligomycin and 100 mM 2-deoxyglucose (2-DG, a glucose analogue that suppresses glycolysis through competitive inhibition of HK [62]) were injected consecutively into the assay plate. Three baseline measurements were taken, 3 response measurements were taken after glucose and oligomycin injection and 2 after 2-DG injection. ECARs were reported as mpH/min normalized against the protein content. Unless specified otherwise, the third measurement of baseline or after addition of each substrate or compound was used to calculate the absolute ECAR values. Glycolytic pathway inhibitors such as 2-DG and oxamate were added as control to estimate the glycolysis-dependent ECAR. Each datum was determined as a minimum in duplicate. Glycolysis was calculated as ECAR after 20 mM glucose addition and glycolytic capacity as ECAR after the addition of 1 µM oligomycin, in both cases ECAR after 2-DG addition were subtracted. Glycolysis was calculated as ECAR_glucose_–ECAR_2-DG_ and glycolytic capacity as ECAR_oligomycin_–ECAR_2-DG._

### 4.9. Statistical Analysis

Data were analyzed by t-test or ANOVA using GraphPad Prism 7. One-way or two-way ANOVA were performed followed by Bonferroni’s Multiple Comparison Test. Unless indicated otherwise, the experiments were performed using three independent culture preparations and in triplicate or quadruplicate. Values are expressed as mean ± SEM. Values with different superscript letters (a, b, c, d and f) indicate significant differences (*p* < 0.05) between groups. Compound and stimuli (androgen, Tip60 and both together) were included in each assay. The graphs were plotted per stimulus in LNCaP cells, in comparison to the basal situation in untreated non-stimulated LNCaP cells.

## 5. Conclusions

In conclusion, sulforaphane and capsaicin decrease proliferation and glycolysis induced by androgen and Tip60, by modulating AR and HIF-1α signaling. These effects were also associated with a decrease in the nuclear Tip60 levels (Supplementary Figure S8A–C). Considering this, we suggest that the protective effect of sulforaphane and capsaicin in LNCaP cells is mediated by Tip60 inhibition. As dietary natural products can be regularly delivered through the diet, sulforaphane and capsaicin could be cost-effective alternatives to promote cancer prevention or to complement pharmacological therapies in prostate cancer treatment. 

## Supplementary Materials

Supplementary materials can be found at https://www.mdpi.com/1422-0067/20/21/5384/s1.
