# Supplementary material for: The Molecular Effects of Sulforaphane and Capsaicin on Metabolism upon Androgen and Tip60 Activation of Androgen Receptor
Source: Int J Mol Sci. 2019 Oct 29;20(21):5384. doi: 10.3390/ijms20215384 (PMC6861939; doi:10.3390/ijms20215384)
Supplement: Supplementary file 1 [file ijms-20-05384-s001.pdf]

Supplementary Figure 1

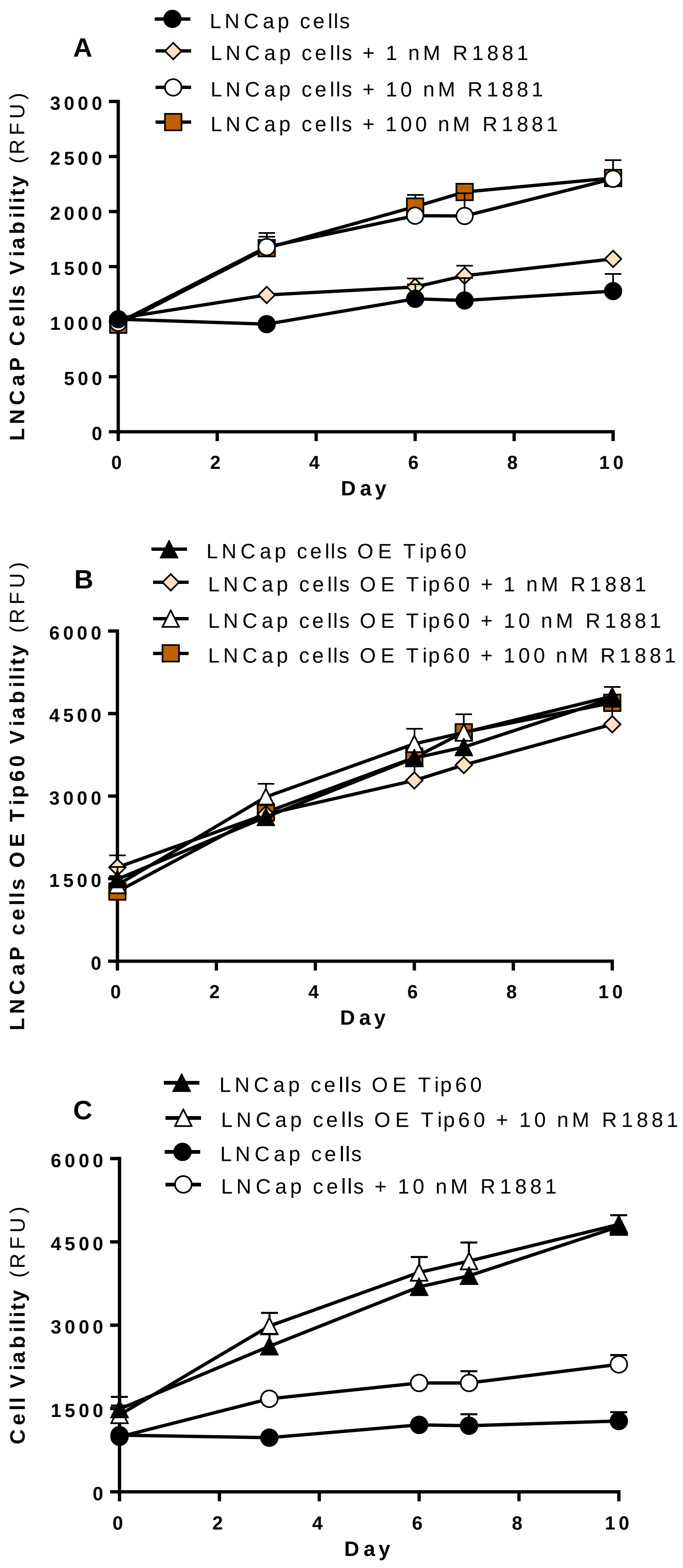

Supplementary Fig. 01. Effect of synthetic androgen (R1881) in the growth of LNCaP cells and LNCaP cells overexpressing Tip60. 10-day cell growth curve of LNCaP cells (A) and LNCaP cells overexpressing Tip60 (B), in the absence or presence of 1, 10 or 100 nM R1881. The effect of 10 nM R1881 in both cell lines is compared (C). Media and androgen were refreshed every 2 days. The cell viability was assessed through their ability to reduce resazurin after 3, 6, 7 and 10 days of cell seeding. Values are expressed as mean ± SEM, from three independent culture preparations, each treatment performed in quadruplicate.

Supplementary Figure 2

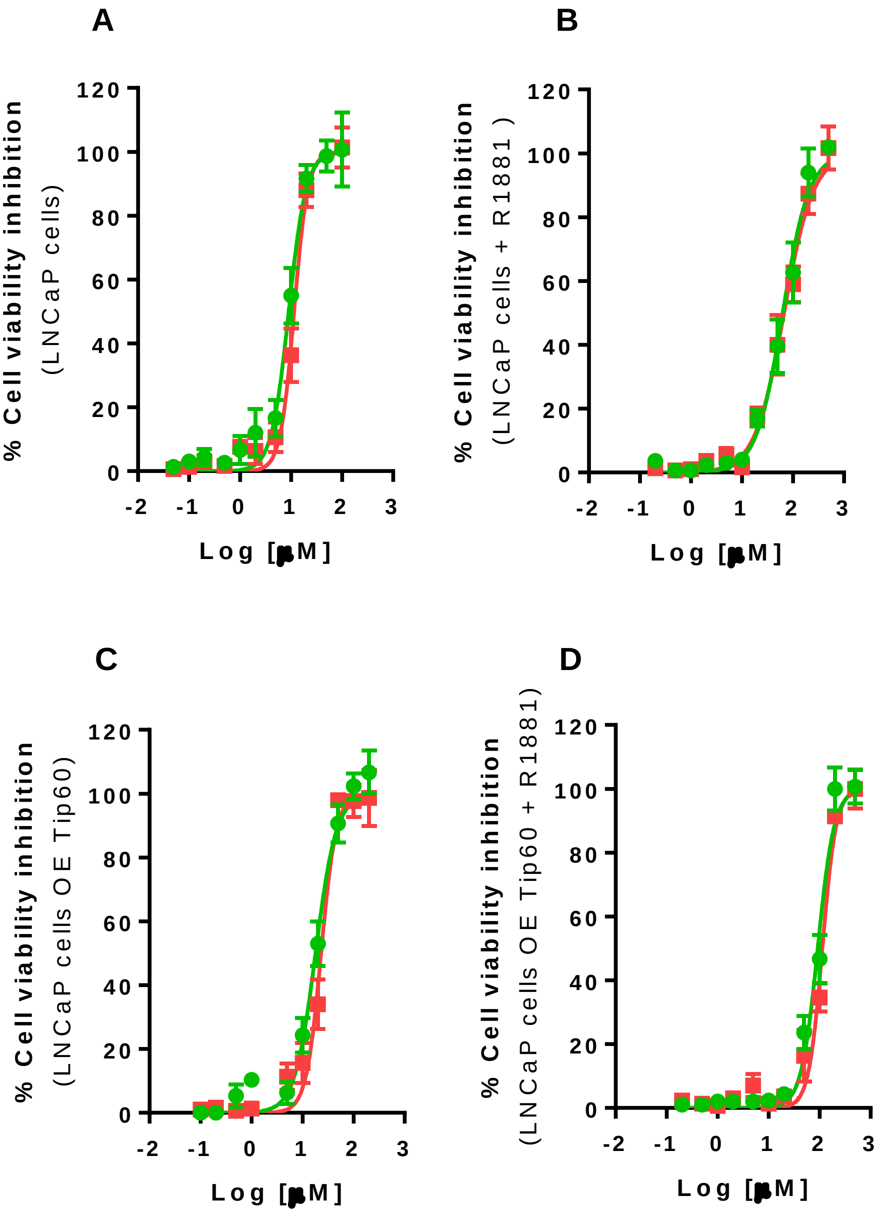

Supplementary Fig. 02. Compounds reduced cell viability of LNCaP cells and LNCaP cells overexpressing Tip60 induced by androgen and Tip60 overexpression, differential effect induced by androgen stimulus. Dose-response effect of sulforaphane (SFN, ■) and capsaicin (CAP, ■) on the viability of (A) LNCaP cells, (B) LNCaP cells + R1881, (C) LNCaP cells overexpressing (OE) Tip60 and (D) LNCaP cells overexpressing (OE) Tip60 + R1881). Compounds were added every 24h for 3 days and R1881 was added at 10 nM for 72h (Scheme 1).

Supplementary Table 1. IC_{50} values of sulforaphane and capsaicin on 72hr proliferation assay in LNCap cells

	LNCaP + STIMULUS (mean ± SEM)			
**TREATMENT**	**NONE**	**10 nM R1881**	**Tip60**	**Tip60 + 10 nM R1881**
**SFN**	9.56 ± 0.35	63.2 ± 3.11	18.0 ± 0.64	92.2 ± 4.21
**CAP**	11.5 ± 0.40	67.0 ± 5.21	23.5 ± 0.74	113 ± 4.87

Supplementary Figure 3

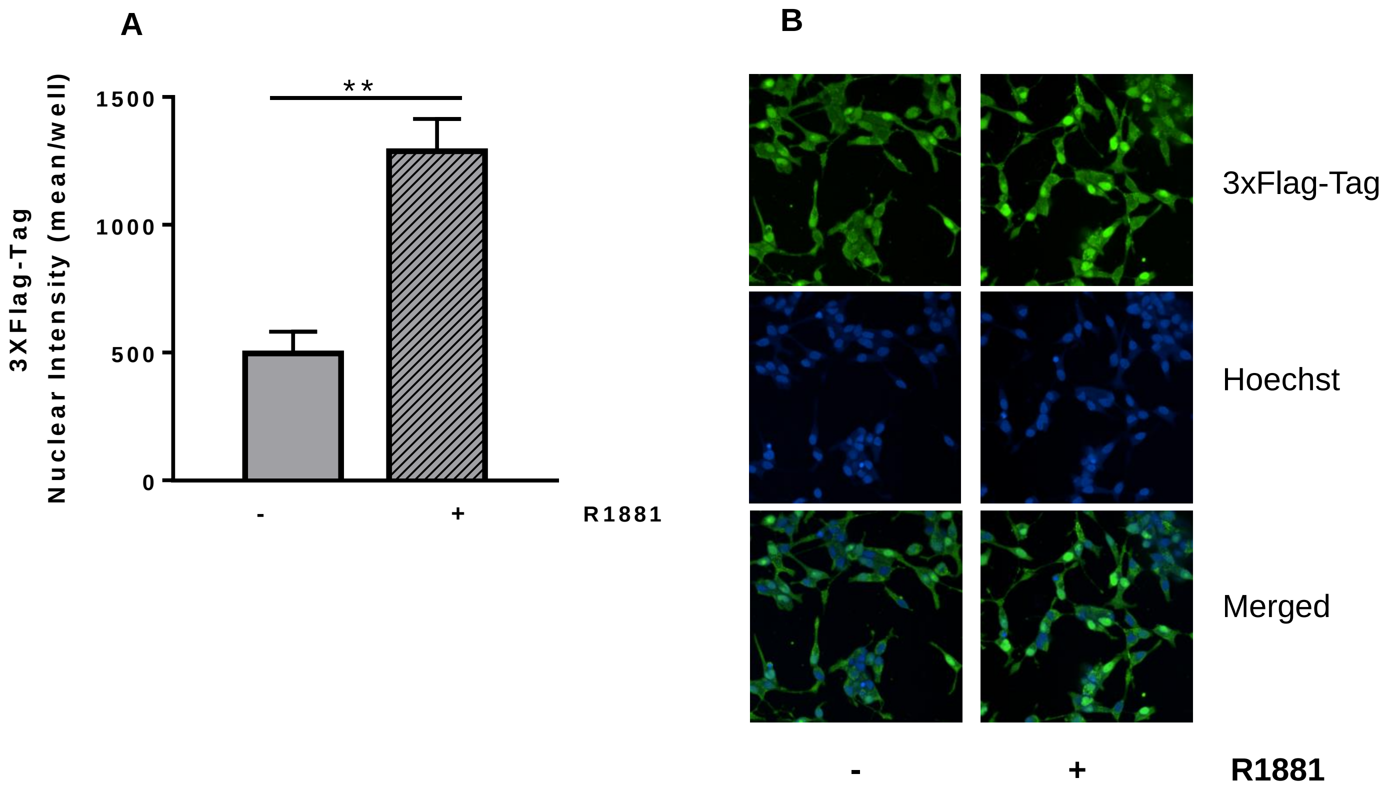

<u>Supplementary Fig. 03. 3xFlag-Tag Tip60 levels are increased by androgen stimulus in LNCaP cells overexpressing Tip60</u>. (A) Nuclear 3xFlag-Tag Tip60 levels and (B) images in transduced LNCaP cells. 3xFlag-Tag Tip60 levels were detected by immunofluorescence using confocal imaging system. Images were acquired with 20x objective. Staining intensity levels in the nucleus were obtained using Harmony software. Nucleus was identified through Hoechst staining. Values are expressed as mean ± SEM, from three independent culture preparations, each treatment performed in quadruplicate. p value comparison from unpaired t-test (**$p<0.01$).

Supplementary Figure 4

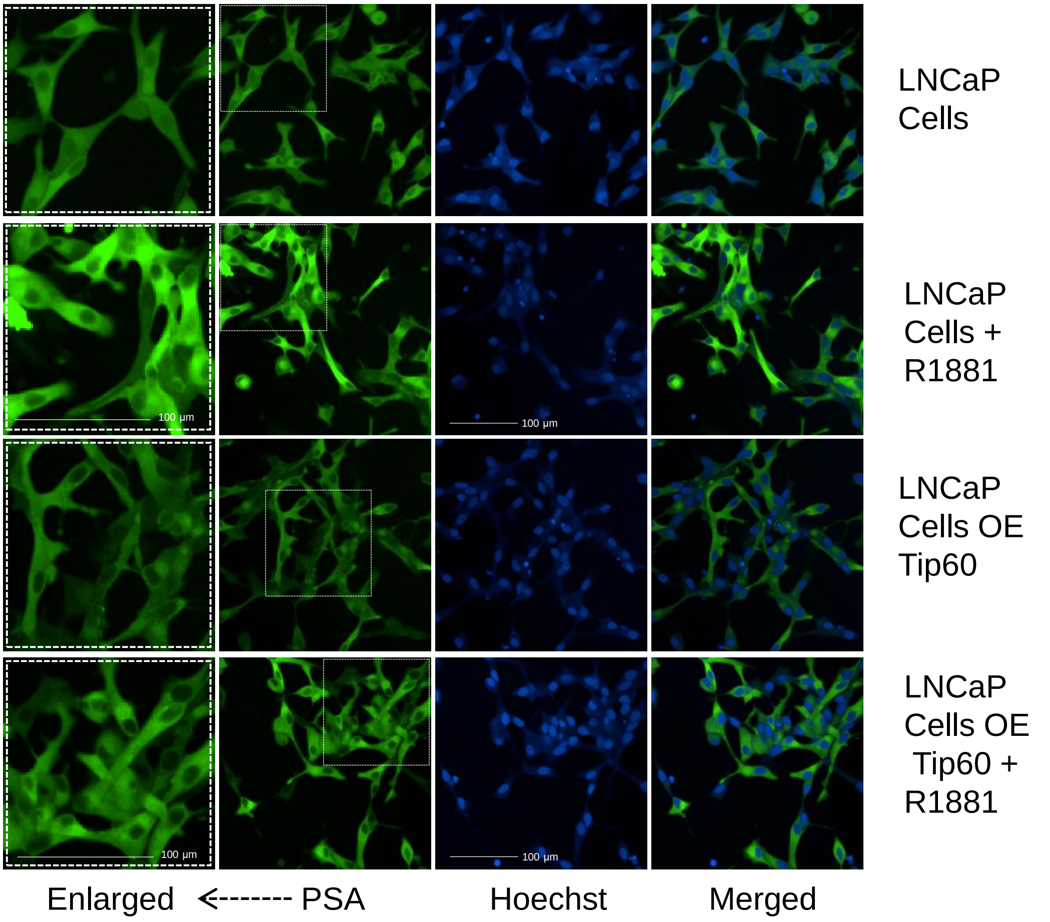

<u>Supplementary Fig. 04. Images of PSA in LNCaP cells.</u> PSA images from LNCaP cells and LNCaP cells overexpressing Tip60, in the absence or presence of androgen (10 nM R1881, 72h) acquired by immunofluorescence using confocal imaging system. Images were acquired with 20x objective. Staining intensity levels in the cytosolic region were obtained using Harmony software. Cytosol was identified through CellMask staining. OE, overexpressing; PSA, prostate specific antigen; R1881, synthetic androgen. Scale is shown as 100 µm.

Supplementary Figure 5

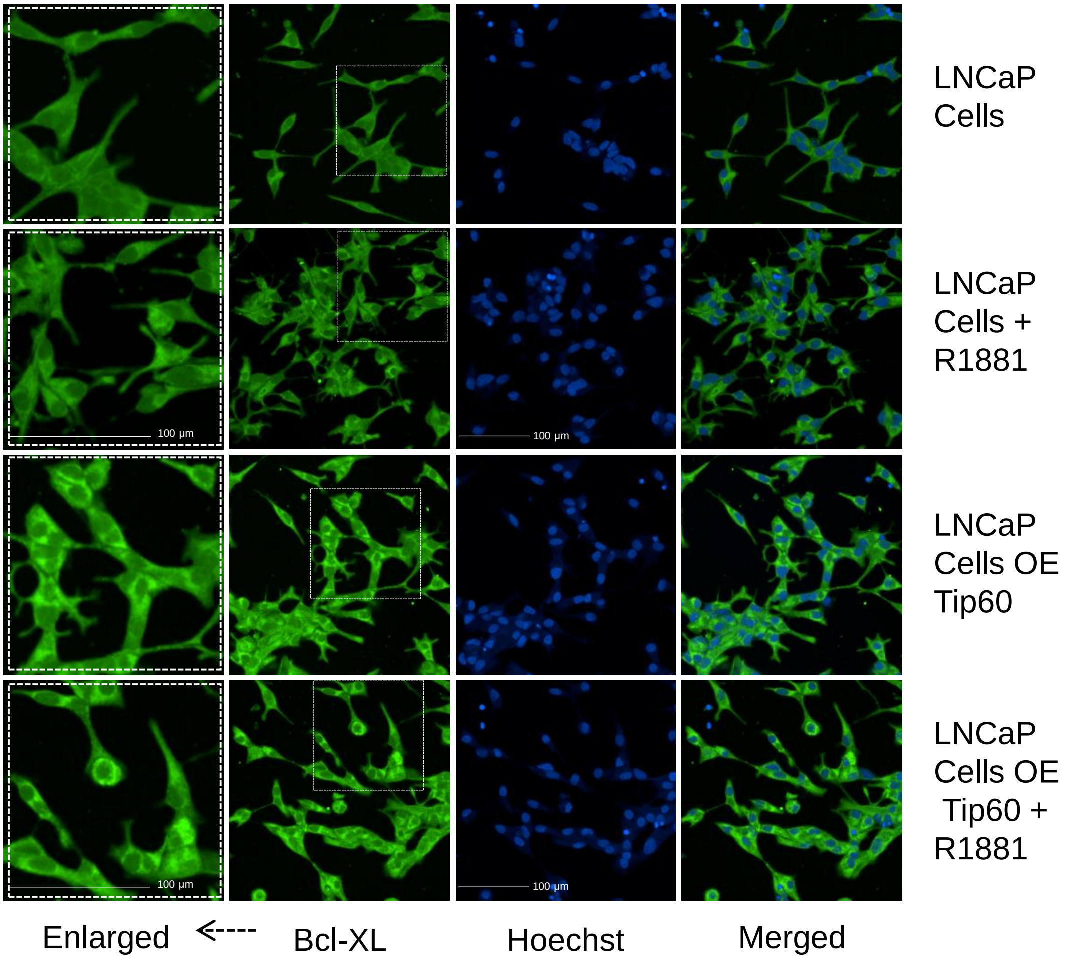

Supplementary Fig. 05. Time course of cell proliferation and images of Bcl-XL in LNCaP cells. Bcl-XL images were detected in LNCaP cells and in LNCaP cells overexpressing Tip60, in the absence or presence of androgen (10 nM R1881, 72h) by immunofluorescence using confocal imaging system. Images were obtained at 20X objective. OE, overexpressing; Bcl-XL, B-cell lymphoma extra-large; OE, overexpressing; R1881, synthetic androgen. Scale is shown as 100 µm.

Supplementary Figure 6

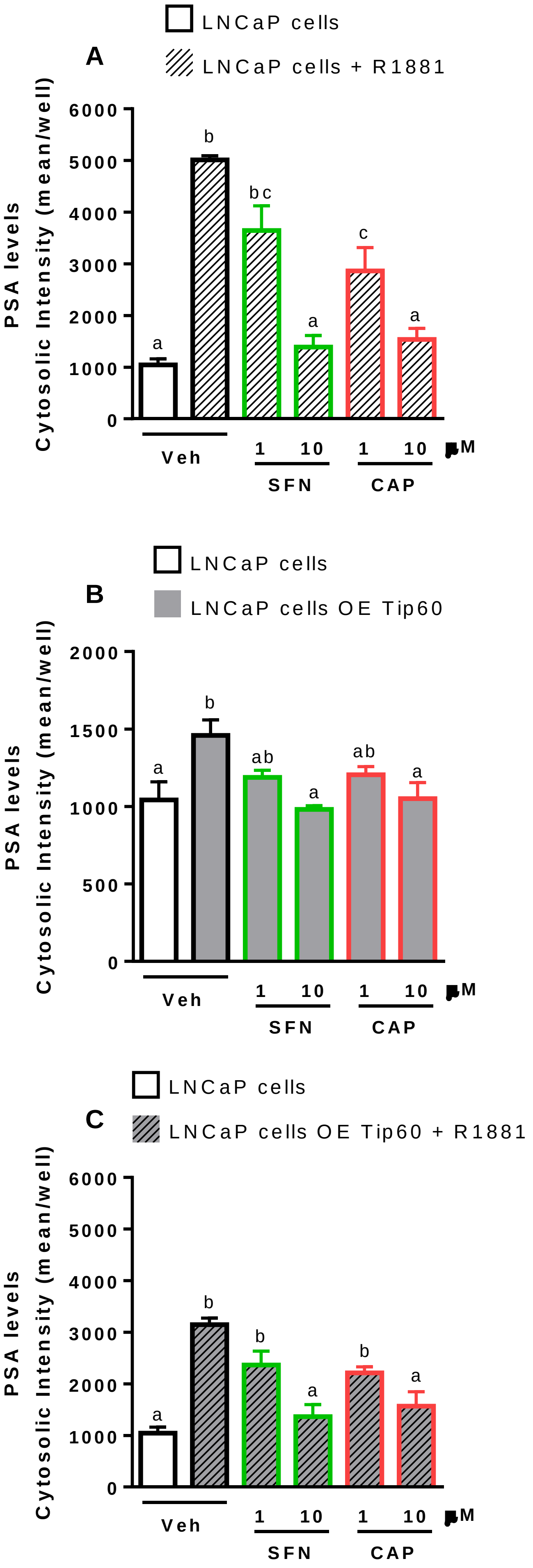

Supplementary Fig. 06. The increase of PSA levels induced by androgen and Tip60 overexpression in LNCaP cells are inhibited by sulforaphane and capsaicin. Cytosolic PSA levels in (A) LNCaP cells + androgen stimulus, (B) LNCaP cells overexpressing Tip60 and (C) LNCaP cells overexpressing Tip60 + androgen stimulus, in comparison to the basal levels of LNCaP cells treated only with vehicle (Veh, 0.4% DMSO). Cells were treated with the compounds added every 24h for 3 days as in scheme 1. PSA levels were detected by immunofluorescence using confocal imaging system. Images were acquired with 20x objective. Staining intensity levels in the cytosolic region were obtained using Harmony software. Cytosol was identified through CellMask staining. Values are expressed as mean ± SEM, from three independent culture preparations, each treatment performed in quadruplicate. For all bars with the same letter, the difference between the means is not statistically significant. Values with different letters indicate significant differences ($p < 0.05$) between bars (one-way ANOVA, Bonferroni post-test). CAP, capsaicin; OE, overexpressing; SFN, sulforaphane.

Supplementary Figure 7

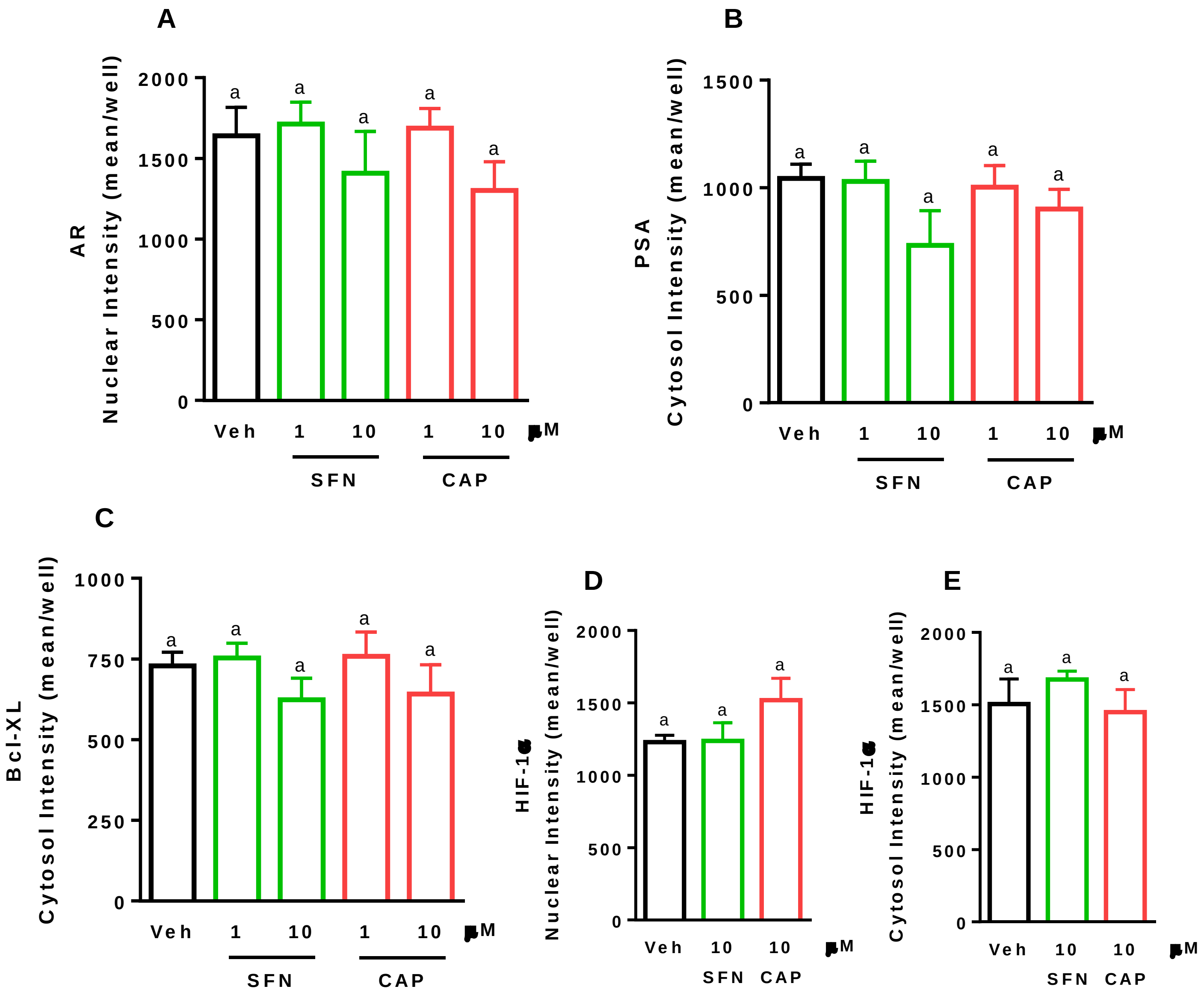

Supplementary Fig. 07. Sulforaphane and capsaicin had no effect on AR, PSA, Bcl-XL, HIF-1α and Tip60 levels in LNCaP cells in the absence of stimuli. (A) Nuclear AR, (B) cytosolic PSA and (C) Bcl-XL, (D) nuclear and (E) cytosolic HIF-1α and (F) Tip60 levels in LNCaP cells. Cells were treated with the compounds, added every 24h for 3 days as in scheme 1. Levels were detected by immunofluorescence using confocal imaging system. Staining intensity levels in the cytosolic region from images acquired with a 20x objective were obtained using Harmony software. Values are expressed as mean ± SEM, from three independent culture preparations, each treatment performed in quadruplicate. For all bars with the same letter, the difference between the means is not statistically significant. Values with different letters indicate significant differences ($p < 0.05$) between bars (one-way ANOVA, Bonferroni post-test). CAP, capsaicin; OE, overexpressing; SFN, sulforaphane, Veh, vehicle (0.4% DMSO)

Supplementary Figure 8

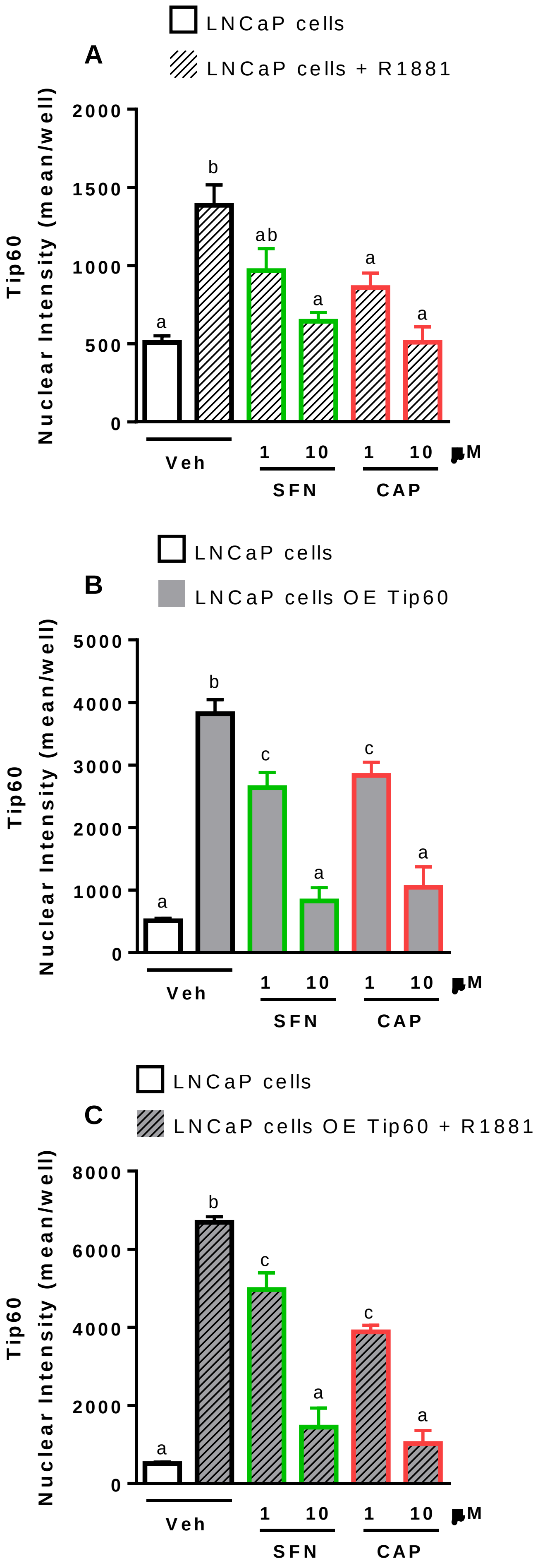

<u>Supplementary Fig. 08. sulforaphane and capsaicin reduced nuclear Tip60 levels in LNCaP cells.</u> Nuclear Tip60 levels in (A) LNCaP cells + androgen stimulus, (B) LNCaP cells overexpressing Tip60 and (C) LNCaP cells overexpressing Tip60 + androgen stimulus, in comparison to the basal levels of LNCaP cells treated only with vehicle (Veh, 0.4% DMSO). Cells were treated with the compounds added every 24h for 3 days as in scheme 1. Tip60 levels were detected by immunofluorescence using confocal imaging system. Images were acquired with 20x objective. Staining intensity levels in the nucleus region were obtained using Harmony software. Nucleus was identified through Hoechst staining. Values are expressed as mean ± SEM, from three independent culture preparations, each treatment performed in quadruplicate. For all bars with the same letter, the difference between the means is not statistically significant. Values with different letters indicate significant differences ($p < 0.05$) between bars (one-way ANOVA, Bonferroni post-test). CAP, capsaicin; OE, overexpressing; SFN, sulforaphane.

Supplementary Table 2. Kinetic assay to evaluate the activity of hexokinase and pyruvate kinase.

ENZYME	REACTION MIX (FINAL CONCENTRATIONS)	INITIATING SUBSTRATE	READOUT
**HEXOKINASE**	60 mM Triethanolamine (pH 7.4), 3.6 mM ATP, 1 mM β-NADP, 8.5 mM $MgCl_2$, 5U/mL Glucose 6-phosphate dehydrogenase (Sigma G8404)	250 mM D-glucose	Reduction of NADP at 340 nm
**PYRUVATE KINASE**	70 mM Imidazole (pH 7.2), 5 mM ATP, 40 mM $MgCl_2$, 50 mM KCl, 0.6 mM β-NADH, 0.5U/mL Lactate dehydrogenase (Sigma L2500)	20 mM phosphoenolpyruvate	Oxidation of NADH at 340 nm
